# Fatty acid composition, antioxidant and antibacterial properties of the microwave aqueous extract of three varieties of *Labisia pumila* Benth

**DOI:** 10.1186/0717-6287-48-9

**Published:** 2015-01-23

**Authors:** Ehsan Karimi, Hawa ZE Jaafar, Ali Ghasemzadeh, Mahdi Ebrahimi

**Affiliations:** Department of Crop Science, Faculty of Agriculture, Universiti Putra Malaysia, 43400, Serdang, Selangor Malaysia; Department of Veterinary Preclinical Sciences, Faculty of Veterinary Medicine, 43400 Serdang, Selangor Malaysia

**Keywords:** Fatty acid composition, Microwave aqueous extraction, Anti-oxidant activities, Anti-bacterial activities

## Abstract

**Background:**

The present study was conducted in order to evaluate the fatty acid profile, anti-oxidant and anti-bacterial activities from the microwave aqueous extract of the leaves of three different varieties of *Labisia pumila* Benth.

**Results:**

The chemical analysis of the extract showed that fatty acids (palmitic, palmitoleic, stearic, oleic, linoleic and α-linolenic) acid as the main components in three varieties of *L. pumila* leaves. Furthermore, the obtained results of the anti-oxidant revealed that *L. pumila* var. *alata* contained higher anti-oxidative activities compared to var. *pumila* and var. *lanceolata*. However, these values were lower than the tested anti-oxidant standards. On the other hand, the aqueous leaf extracts in all three varieties of *L. pumila* were also found to inhibit a variable degree of antibacterial activities against eight bacteria (four Gram-positive and four Gram-negative bacteria).

**Conclusions:**

In this study, it was observed the leaves of three varieties of *L. pumila* exhibited variable patterns of fatty acids and the microwave aqueous extraction possess anti-oxidant and anti-bacterial activities.

## Background

Anti-oxidant is a chemical substance extremely useful due to prevent or delay the formation of free radicals and lipid peroxidation in the animals and human bodies, two main causes of animals and human disease and aging [1]. It helps us ward off many kinds of disease related to lungs, kidneys, heart, cardiovascular system, muscle and brain, and it helps to retard the aging process [2].

Fatty acids are molecules typically found attached to other compounds such as sugars, glycerol or phosphate head groups to form lipids. Lipids are necessary components of cell structures, for example for cell membranes, which are main components' of phospholipids, and energy stores that are often composed of triglycerides. Fatty acids are released from lipids, typically by the action of enzymes, to become free fatty acids, which have vast and potent biological activities [3]. The biological activities of free fatty acids have roles in host defenses against potential opportunistic or pathogenic microorganisms. An important aspect of this is growth inhibition or the quick destroying of bacteria. several studies for understanding the mechanism of the anti-bacterial effects of different fatty acids from a wide range of biological sources such as algae, animals and plants have been done by several researchers [3, 4]. Indeed, fatty acids are normally identified as the active ingredients in ethnic and herbal medicines [5, 6]. Tropical and subtropical plants are perceived to produce a large variety of phytochemicals or secondary metabolites and possess a wide range of cancer preventive. Most of these secondary metabolites are isolatedfrom wild or cultivated plants because their chemical synthesis is either extremely difficult or economically not available due to their highly complex structures and specific stereo-chemical requirements of the compounds [7]. *Labisia pumila* Benth. (Myrsinaceae) is a wild forest herb and reported contain several bioactive compounds, which include phenolics, triterpenoids (e. g. phytosterols, saponins, sapogenins, non-steroidal and triterpenoids) and peptides [8]. The chemistry of many species of this family is little known of traditional uses to treat various ailments [9]. This study was aimed to investigate the total phenol and flavonoid content, fatty acid profiling, anti-oxidant activity and anti-bacterial properties of aqueous extracts of three varieties of medicinal plant, *L. pumila* Benth. obtained from microwave extraction.

## Results

### Total phenolics (TP) and flavonoids (TF) compounds

The total phenolic and flavonoid contents results from the study showed that in general the leaves of *L. pumila* var. *pumila* had higher total flavonoids content than var. *alata* and var*. lanceolata* But, the aqueous leaf extract of *L. pumila* var*. alata* contained higher total phenolics than var. *pumila* and var. *lanceolata* (Table 1). Total phenolics and flavonoids content of leaves in all three varieties of *L. pumila* were significantly different with each other.

**Table 1:** **Total phenolics and flavonoids content of the leaves aqueous extract of three varieties of** ***Labisia pumila*** **Benth (Mean ± SEM; n = 3)**

Leaf extract	Phenolic content ^1^	Flavonoid content ^2^
*Alata*	3.07 ± 0.28^a^	1.39 ± 0.11^b^
*Pumila*	2.96 ± 0.21^b^	1.94 ± 0.15^a^
*Lanceolata*	2.78 ± 0.24^c^	1.35 ± 0.13^c^

^1^mg gallic acid equivalent/g DW; ^2^mg rutin equivalent/g DW; Results are means of three replicates ± standard error. Means with the different letters are significantly different from each other at *P* < 0.05.

### Analysis of fatty acid profiling

The fatty acid composition of the *L. pumila* leaves extract in different varieties was presented in Table 2. The most abundant fatty acid present in the aqueous extract of *Labisia* was (C16:0 and C18:2n-6), which accounted for approximately 24.58-28.21% and 24.49-28.84% of total identified fatty acids. The palmitic acid (C16:0) was highest in *alata* variety when compared to other varieties. The proportion of leaves extract fatty acids having 18 carbons was quite consistent across the three varieties, averaging between 69.51 to 71.53% (Table 2). Mean concentrations of C18:0, C18:1n-9, C18:2n-6, and C18:3n-3 were 4.36, 18.73, 27.52, and 21.20%, respectively. On the other hand, C18:3n-3 in *Lanceolata* variety showed a significantly higher amount compared to other varieties. The varieties showed significant (*P* < 0.05) effects on C18:3n-3 in the *Labisia pumila* leaves.

**Table 2:** **Fatty acid profile of microwave aqueous extract of three varieties of** ***Labisia pumila*** **Benth (Mean ± SEM; n = 3)**

	***Alata***	***Pumila***	***Lanceolata***
**C16:0**	28.21 ± 1.31^a^	27.05 ± 3.50^ab^	24.58 ± 0.54^b^
**C16:1**	2.29 ± 1.02	1.43 ± 0.26	1.05 ± 0.29
**C18:0**	4.67 ± 0.34	4.64 ± 0.38	3.76 ± 0.57
**C18:1**	18.83 ± 0.08	19.67 ± 0.85	17.68 ± 0.42
**C18:2 n-6**	24.99 ± 0.80	28.73 ± 2.44	28.84 ± 0.20
**C18:3 n-3**	21.01 ± 2.29^ab^	18.50 ± 0.86^b^	24.08 ± 0.21^a^
^**1**^ **Total saturated**	32.88 ± 1.51	31.68 ± 3.88	28.34 ± 0.73
^**2**^ **Total monoenes**	21.12 ± 0.98	21.09 ± 0.61	18.73 ± 0.53
^**3**^ **Total PUFA n-3**	21.01 ± 2.29^ab^	18.50 ± 0.86^b^	24.08 ± 0.21^a^
^**4**^ **Total PUFA n-6**	24.99 ± 0.80	28.73 ± 2.44	28.84 ± 0.20
^**5**^ **Total polyunsaturated**	46.00 ± 2.81^b^	47.23 ± 3.28^b^	52.92 ± 4.25^a^
**PUFA n-6 : PUFA n-3**	1.19 ± 0.15	1.55 ± 0.06	1.20 ± 0.01
**Polyunsaturated: saturated**	1.41 ± 0.13	1.57 ± 0.32	1.87 ± 0.05

^1^Total Saturated = sum of C16:0 + C18:0.

^2^Total Monoenes = sum of C16:1 + C18:1n-9.

^3^Total n-3PUFA = sum of C18:3n-3.

^4^Total n-6PUFA = sum of C18:2n-6.

^5^Total Polyunsaturated fatty acid = C18:2n-6+ C18:3n-3.

Values with different superscripts between rows differ significantly at *P* <0.05.

### Anti-oxidant activity (ABTS and NO scavenging)

**Figure 1:**
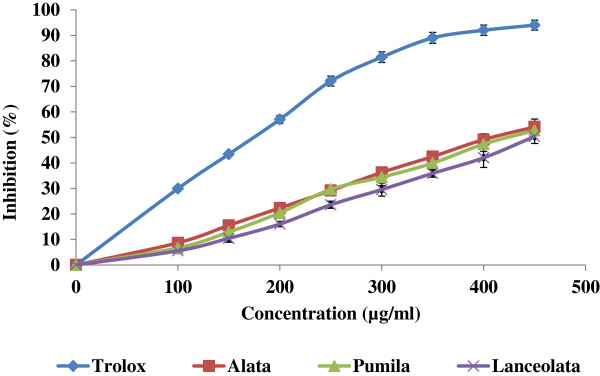
**ABTS radical cation-scavenging of three varieties of** ***L. pumila*** **extracts (** ***alata*** **,** ***pumila*** **and** ***lanceolata*** **) and trolox at different concentrations.** Each value represents mean ± SEM of three replicates.

**Figure 2:**
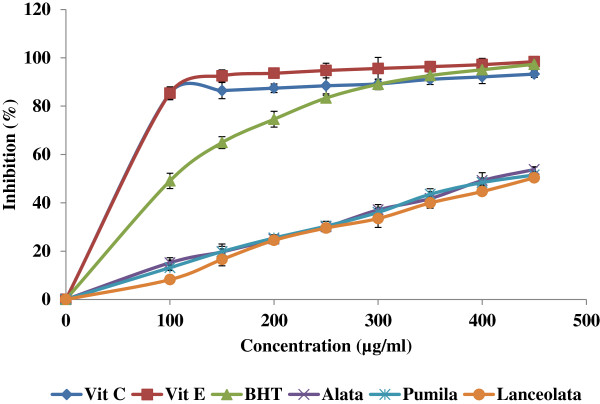
**Nitric oxide scavenging activity of three varieties of** ***L. pumila*** **extracts (** ***alata*** **,** ***pumila*** **and** ***lanceolata*** **) and vitamins at different concentrations.** Each value represents mean ± SEM of three replicates.

Figures 1 and 2 show the anti-oxidant activities of aqueous extracts obtained from *Labisia pumila* var. *alata*, *pumila* and *lanceolata* in the reactions with ABTS radical and nitric oxide respectively. The obtained results revealed that the *L. pumila* var. *alata* contained higher antioxidative activities compared to var. *pumila* and *lanceolata*. However, these values were lower than the tested anti-oxidant standards. The aqueous extracts inhibited the free radicals in a dose dependent manner. The IC_50_ concentrations (Table 3) showed significant (*P* < 0.05) differences in ABTS and NO scavenging activity among samples, where *L. pumila* var. *alata* showed the lowest value, followed by *pumila* and *lanceolata*.

**Table 3:** **The IC50 values of extracts and standards in ABTS and nitric oxide scavenging activities (Mean ± SEM; n = 3)**

Samples	IC _50_ (μg/mL)	
	ABTS radical scavenging activity	NO scavenging activity
*L. pumila* var. *alata*	399.02 ± 1.12^c^	411.53 ± 1.65^c^
*L. pumila* var. *pumila*	424.57 ± 1.87^b^	425.87 ± 1.52^b^
*L. pumila* var. *lanceolata*	448.20 ± 2.15^a^	449.29 ± 2.03^a^
Vitamin C	-	59.91 ± 3.42^f^
Vitamin E	-	62.6 ± 2.62^e^
BHT	-	118.73 ± 1.73^d^
Trolox	187.47 ± 1.01^d^	-

Means in the same column with the different superscripts are significantly different at *P* < 0.05.

### Anti-bacterial activity determination

The result obtained from anti bacterial assays is presented in Table 4 at concentration of 300 μg/disc. The extracts of leaves in all three varieties of *L. pumila* were also found to inhibit a variable degree of antibacterial activities against eight bacteria (four Gram-positive and four Gram-negative bacteria). The inhibition from those extracts showed low inhibition compared to kanamycin, as the positive control. Kanamycin as a standard antibiotic showed high inhibition zones at a concentration of 1 μg/disc. As a comparison among three varieties, *L. pumila* var. of *pumila* exhibited higher inhibition zone with diameters ranging from 0.37 - 0.75 cm against (*P. aeruginosa* and *B. subitilis*), compared to *L. pumila* var. *alata* and *lanceolata*. Meanwhile, *L. pumila* var. *alata* exhibited higher inhibition zone with diameters ranging from 0.4 - 1.03 cm against (*Pseudomonas aeruginosa* and *Escherichia coli*) compared to *L. pumila* var. *pumila* and var. *lanceolata*. From the results, it can be concluded that Gram-positive bacteria were found to be more sensitive against the extracts than Gram-negative bacteria.

**Table 4:** **Inhibition zones of aqueous leaf extracts of three varieties of** ***Labisia pumila*** **against pathogenic bacteria at concentration of 300 μg/disc (Mean ± SEM; n = 3)**

Sample	Inhibition zone (cm)			
	Gram-positive bacteria			
	***B. subitilis***	***S. aureus***	***B. cereus***	***M. luteus***
*Alata*	0.72 ± 0.11^b^	0.65 ± 0.15^c^	0.75 ± 0.13^b^	0.55 ± 0.06^b^
*Pumila*	0.75 ± 0.23^b^	0.70 ± 0.13^b^	0.73 ± 0.25^b^	0.50 ± 0.17^b^
*Lanceolata*	0.65 ± 0.46^c^	0.55 ± 0.07^d^	0.60 ± 0.16^c^	0.40 ± 0.22^c^
Kanamycin	1.15 ± 0.05^a^	0.95 ± 0.03^a^	1.02 ± 0.09^a^	0.91 ± 0.05^a^
	**Gram-negative bacteria**			
	***E. coli***	***P. aeruginosa***	***E. aerogenes***	***K. pneumonie***
*Alata*	0.90 ± 0.15^b^	0.40 ± 0.23^b^	0.63 ± 0.07^b^	0.60 ± 0.09^bc^
*Pumila*	0.85 ± 0.09^c^	0.37 ± 0.17^bc^	0.45 ± 0.15^c^	0.65 ± 0.25^b^
*Lanceolata*	0.78 ± 0.12^d^	0.30 ± 0.11^c^	0.40 ± 0.19^cd^	0.55 ± 0.33^c^
Kanamycin	1.30 ± 0.03^a^	0.92 ± 0.05^a^	1.05 ± 0.05^a^	1.20 ± 0.11^a^

Means with different superscripts within column are significantly different (*P <* 0.05).

## Discussion

Natural phytochemicals including flavonoid and phenolic compounds and fatty acids are major bioactive compounds known to be beneficial against many diseases and have been reported to possess a wide range of biological effects like anti-oxidant and antibacterial activities [10]. Phenolic and flavonoid compounds, important phytochemicals, are present in vegetables, fruits and cereal grain. These secondary metabolites are natural anti-oxidants that have multiple biological effects and play an important role in the defense against cardiovascular disease, aging and cancer [11]. The results of the present study showed that aqueous leaves extract obtained from microwave extraction have anti-oxidant potentials and antibacterial properties. From the results it can be concluded that *L. pumila* var. *lanceolata* possess consistently the lowest values of total phenolics and total flavonoids contents, anti-oxidant and antibacterial activities when compared to the other two varieties, which are popularly researched on. This is maybe due to the variation of secondary metabolite and fatty acid content present in the extracts. For examples apigenin [12] naringin and naringenin [13], quercetin, 3-O-methylquercetin and various quercetin glycosides [13], gallic acid and pyrogallol [14] were identified to possess high anti-oxidant and antibacterial activities. It was found that the aqueous extract of *Labisia pumila* leaves contains fatty acids (palmitic, palmitoleic, stearic, oleic, linoleic and α-linolenic) with the latter as main component. This is in the first report of *Labisia pumila* leaves fatty acid profiles. The fatty acids are said to exhibit antibacterial activity [3]. Fatty acids can act as anionic surfactants and have antibacterial and antifungal properties at low pH [15]. In addition to being selective against Gram-positive organisms [16] by targeting the structure and function of bacterial cell walls and membranes. These components may be the contributing factors to the effect demonstrated by the aqueous extract of *Labisia pumila*. The similar studies showed thatpalmitic, stearic, oleic, linoleic and linolenic acids are well known to have antibacterial activities [5]. Our study undoubtedly confirms that the leaves extract of *Labisia pumila* contains a higher relative percentage of the above-mentioned fatty acids that has potential anti-oxidant and anti-bacterial principle for clinical application.

## Conclusions

Throughout history, natural products have afforded a rich source of compounds that have found many applications in the fields of medicine, pharmacy and biology [17]. The present study elucidates the potentials effects of the antioxidant and antibacterial properties of the *Labisia Pumila* leaves obtained from the microwave extraction. These antioxidant and antimicrobial activities of *L. pumila* could be attributed to various fatty acids and phytochemical constituents (flavonoid, phenolic) present in the aqueous leaves crude extract. The leaf is the main source of antimicrobial and anti-oxidants, which have shown to posses radical scavenging activities and reducing potential.

## Methods

### Chemicals

Methanol, hydrochloric acid, Folin-Ciocalteu reagent, sodium carbonate, aluminium chloride, sodium hydroxide, ascorbic acid, alpha-tocopherol, butylatedhydroxytoluene (BHT), dimetylsulfoxide (DMSO), KOH and trifluoride (BF_3_) were purchased from Fisher Scientifics, USA. The other chemicals used in this study were bought from Merck.

### Plant material

Seedlings of *Labisia pumila* varieties *alata, pumila*, and *lancelota* were, respectively, collected from places of origin at Sungkai, Perak; Hulu Langat, Selangor, and Kota Tinggi, Johore, and raised under similar glasshouse condition for 18 months before use in the study. The GPS location details were 3° 0'35.27"N latitude and 101°4219.38"E longitude. Healthy and uniform seedlings in term of leaf numbers were selected from the three varieties. The leaves of three varieties of *Labisa pumila* Benth. were cleaned, separated, and freeze dried for further analysis.

### Microwave Assisted Extraction (MAE)

MAE was performed on microwave apparatus using a closed vessel system with pressure (ETHOS^®^ T Microwave digestion/extraction system, Milestone Co., Italy) based on the method described by Xiao et al. [18] with some modification. One gram of leaf part of three varieties of *Labisia pumila* was weighed using a clean aluminum container, then transferred into the vessel of the Ethos E Microwave Extraction System and extracted with 30 ml of water as solvent for 2 min (p = 750 w). The extraction temperature was applied to 80°C. After extraction, the vessels were allowed to cool at room temperature before opening. Then the extracts were filtered and stored in refrigerator.

### Total phenolics determination

For total phenol determination, briefly 0.5 ml of each methanolic extract, 2 ml of 7.5% sodium carbonate and 2.5 ml Folin-Ciocalteu reagent were mixed together. The mixture was then vortex and incubated for 90 min at room temperature [19]. The absorbance was read using a spectrophotometer (Novaspec II Visiblespectro, Japan) at 765 NM. The total phenol results were expressed as mg gallic acid equivalents (GAE)/g dry weight (DW).

### Total flavonoids determination

For total flavonoid compounds 0.1 ml of methanolic extracts was added to 0.3 ml sodium nitrite (5%) and incubated for 5 min at room temperature, then 0.3 ml 10% (w/v) AlCl3 and 2 ml 1 N NaOH was added and the total volume was made up to 5 ml with distilled water [19]. The absorbance was measured at 510 nm by using visible spectrophotometer (Novaspec II Visiblespectro, Japan) at 510 nm. The results were expressed as mg rutin equivalents/g DW.

### Fatty acid profile determination

The total fatty acids of the leaves were extracted according to the method of Folch et al. [20] with some modifications as described by Ebrahimi et al. [21], using chloroforms: methanol 2:1 (v/v) which contained butylated hydroxy toluene to prevent the oxidation during fatty acid extraction. Extracted fatty acids Trans methylated to the fatty acid methyl esters (FAME) using KOH in methanol and 14% boron trifluoride (BF_3_) in methanol. The FAME were separated using gas liquid chromatography (Agilent 7890A), using a Supelco SP 2560 capillary column of 100 m × 0.25 mm ID × 0.2 μm film thickness (Supelco, Inc., Bellefonte, PA, USA). One microliter was injected into the gas chromatography, equipped with an injector and a flame ionization detector. The nitrogen was the carrier gas at a flow rate of 1.2 ml/min. The split ratio was 1: 10. The temperature of the injector was 250°C and the detector temperature was 270°C. The column temperature program started runs at 150°C, for 2 min, warmed to 158°C at 1°C/min, held for 28 min, warmed to 220°C at 1°C/min, and then held for 20 min. A reference standard (C4-C24 methyl esters; Sigma-Aldrich, Inc., St. Louis, Missouri, USA), was used to determine correction factors for the determination of individual fatty acid composition. The data are expressed as g/100 g of detecting total identified fatty acids.

### Anti-oxidant activity assay

#### Nitric oxide (NO) scavenging activity

The nitric oxide (NO) scavenging activity of each plant extract was determined by the method of Tsai et al. [22]. Vitamin C, BHT and α-tocopherol were used as controls. The NO scavenging activity was calculated according to the formula: [(A0 - A1)/A0] × 100%; where A0 was the absorbance of the control reaction and A1 was the absorbance in the presence of the sample.

#### ABTS radical cation-scavenging

The ABTS was evaluated by Giao et al*.*[23] method. ABTS was dissolved in water, to a 7 mm concentration. ABTS radical cation (ABTS.^+^) was produced by reacting ABTS stock solution with 2.45 mM K_2_S_2_O_8_ and allowing the mixture to stand at room temperature (dark place) overnight before utilization.

### Anti-bacterial activity assay

The antibacterial assay of the leaf extracts of three varieties of *L. pumila* was carried out by the disc diffusion method as described by Boussaada et al*.*[24] against *Staphylococcus aureus* S1431, *Escherichia coli* E256, *Pseudomonas aeruginosa* PI96*, Micrococcus luteus*, *Klibsiella pneumonia* K36, *Bacillus subtilis* B145*, Bacillus cereus* B43 and *Enterococcus aeruginosa*. All the bacteria were purchased from the Institute of Malaysian Research (IMR) and maintained in the department of Microbiology, Faculty of Biotechnology and Biomolecular Sciences, Universiti Putra Malaysia. In this assay, the positive control without extracts (solvent) and reference control used kanamycin as the standard antibiotic agent. The extracts inhibitions were corrected based on positive control values. The experiments were run in triplicate.

### Statistical analysis

All data are presented as means (± SEM) of at least three replicates (*n* = 3). The total phenolic and flavonoid contents, fatty acid, anti-oxidant and anti-bacterial properties were analyzed using analysis of variance (ANOVA) with the Statistical Analysis System (SAS) Version 9.1 (SAS Institute, Cary, NC). Significant differences among means from triplicate analyses (p < 0.05) were determined by Duncan’s Multiple Range Test. The level of significance was set at p < 0.05 for all statistical tests.
